# Characterization of Oral *Candida* spp. Biofilms in Children and Adults Carriers from Eastern Europe and South America

**DOI:** 10.3390/antibiotics12050797

**Published:** 2023-04-22

**Authors:** Anelise Maria Costa Vasconcelos Alves, Beatriz Oliveira Lopes, Ana Caroline Rocha de Melo Leite, Gabriela Silva Cruz, Érika Helena Salles de Brito, Laritza Ferreira de Lima, Lucia Černáková, Nuno Filipe Azevedo, Célia Fortuna Rodrigues

**Affiliations:** 1 Institute of Health Sciences, University of International Integration of Af-ro-Brazilian Lusophony, Av. da Abolição, 3-Centro, Redenção 62790-000, Ceará, Brazil; 2 LEPABE—Laboratory for Process Engineering, Environment, Biotechnology and Energy, Faculty of Engineering, University of Porto, Rua Doutor Roberto Frias, 4200-465 Porto, Portugal; 3 ALiCE-Associate Laboratory in Chemical Engineering, Faculty of Engineering, University of Porto, Rua Doutor Roberto Frias, 4200-465 Porto, Portugal; 4 Laboratory of Oocytes and Preantral Follicles Manipulation—LAMOFOPA, Post-Graduate Program in Veterinary Science, Faculty of Veterinary Medicine, State University of Ceará—UECE, Av. Doutor Silas Munguba, 1700, Campus do Itaperi, Fortaleza 60714-903, Ceará, Brazil; 5 Department of Microbiology and Virology, Faculty of Natural Sciences, Comenidus University in Bratislava, Ilkovičova 6, 842 15 Bratislava, Slovakia; 6 TOXRUN—Toxicology Research Unit, Cooperativa de Ensino Superior Politécnico e Universitário—CESPU, 4585-116 Gandra PRD, Portugal

**Keywords:** antifungals susceptibility, *Candida* species, aging, oral candidiasis, biofilm, resistance

## Abstract

Background: *Candida albicans* and non-*Candida albicans Candida* species (NCACs) are known to colonize and invade various tissues, including the oral mucosa. In this work, we aimed to characterize mature biofilms of several *Candida* spp. clinical isolates (*n* = 33) obtained from the oral mucosa of children, adults, and elders of Eastern Europe and South America. Methods: Each strain was evaluated for its capacity to form biofilms in terms of total biomass using the crystal violet assay and for matrix components production (proteins and carbohydrates) using the BCA and phenol-sulfuric tests, respectively. The effect of different antifungals on biofilm formation was studied. Results: in the children’s group, a predominance of *C. krusei* (81%) was observed, while, among adults, the main species was *C. albicans* (59%). Most strains showed a reduced response to antimicrobial drugs when in biofilm form (*p* < 0.01). Moreover, it was observed that strains isolated from children produced more matrix, with higher levels of protein and polysaccharides. Conclusions: children were more likely to be infected by NCACs than adults. More importantly, these NCACs were able to form biofilms richer in matrix components. This finding is of clinical importance, particularly in pediatric care, since stronger biofilms are highly associated with antimicrobial resistance, recurrent infections, and higher therapeutic failure.

## 1. Introduction

Candidiasis has been increasing in recent decades, mainly due to the growing number of individuals with immunocompromised conditions and the higher antifungal resistance of *Candida* spp. [1]. *Candida* spp. are common colonizers in the oral cavity, vagina, and skin, but it is possible that they might be located in the endocardium and meninges. Systemic infections are mainly observed in adults, however, the clinical features of disseminated disease in adults have only been recently described [2].

In the oral cavity, in which *Candida* spp. is the most common fungus, oral candidiasis (OC) is particularly critical due to its high prevalence in all ages and genders [3]. OC is characterized by oral discomfort, pain, burning sensation, parageusia, and aversion to food [1,4]. It is usually associated with age (childhood or older), smoking, diabetes mellitus, nutritional disorders, endocrinopathies, immunosuppressive conditions, and malignancies [4]. Nevertheless, and importantly, OC can also be observed in 60% of the healthy population and non-immunocompromised individuals [5].

It is known that pathogenic mechanisms of *Candida* spp. depend upon both host conditions and *Candida* virulence factors [6]. Thus, healthy members of the population presenting candidiasis might host *Candida* spp. with virulence factors that are able to switch to the pathogenic form even in healthy immune systems [6]. One of the most important virulence factors of the *Candida* spp. is its ability to form biofilms, which are difficult to control, resulting in chronic or recurrent infections [6]. Biofilms are a sessile community of microorganisms adhered to biotic or abiotic surfaces and embedded in an extracellular polymeric substance (EPS) that forms a matrix [7,8]. This extracellular matrix plays a key role in antimicrobial resistance by preventing antibiotic penetration, avoiding phagocytosis through immune system cells, acting as physical barrier to environmental changes, and promoting microbial adhesion to biotic and abiotic surfaces. The matrix is also responsible for the structural organization of biofilms, forming water channels and allowing intercellular interactions [7,9]. Its composition consists of exopolysaccharides, nucleic acids (eDNA and eRNA), proteins, lipids (e.g., ergosterol), and other biomolecules [8,9]. Biofilm matrix composition is directly associated to the pathogenicity of species/strains, as well as to antifungal drug resistance. 

This study might improve our understanding on how to better treat each species/strain according to a species’ biofilm profile. In addition, the colonization of *Candida* spp. biofilms in different age groups is still poorly explored. Hence, in order to better understand *Candida* species’ oral biofilm formation and their effects in different age groups from different continents, we isolated strains of *C. albicans* and NCACs from Eastern Europe and South America and analyzed the biofilms in terms of chemical composition and antifungal drug susceptibility.

## 2. Results

### 2.1. Demographic Data of Study Population and Ethical Aspects

Participants (*n* = 31) were divided into three groups: children (1–12 years), adults (13–59 years), and elders (+60 years). The average age of participants from the children, adults, and elders’ groups was 3.9±1.97, 34±11.61, and 70±2.83 years old, respectively. Most of the participants in the children’s group were female, while adults and elders were predominantly male. In the children’s group, a predominance of *C. krusei* (81%) was observed, while among adults and elders, the main species was *C. albicans* (63% and 67%, respectively) (See Appendix A).

It is relevant to note that, during the isolation process, only one species was isolated from each person. This could probably be a limitation of the isolation technique itself. CHROMagar *Candida* screening was routinely performed in order to confirm that the isolate was not contaminated (common protocol).

### 2.2. pBiofilm Formation of Candida *spp.*

**Figure 1:**
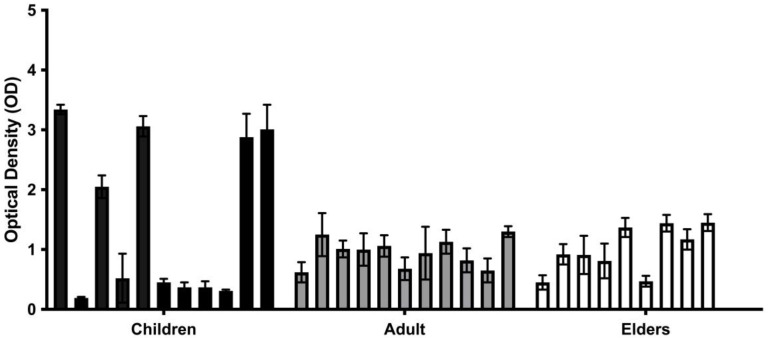
In vitro biofilm production of clinical isolates from children, adults, and elders. The black and grey bars are each from different age groups. Biofilm was quantified through staining with crystal violet (OD_570_) after 48 h of incubation. Each value is the average of three independent experiments conducted in triplicate.

We measured the total biomass of *Candida* spp. biofilms formed by strains isolated from the oral mucosa of the participants of this study through crystal violet (CV) staining (Figure 1). Results showed that all strains are moderate or strong biofilm formers, and the most-isolated *Candida* spp. in children had a higher biofilm mass than the one from the adult group. *C. krusei* BC06 presented the highest biofilm biomass (2.14 Abs/cm^2^ ± 0.08), followed by *C. krusei* BC18 (1.95 Abs/cm^2^ ± 0.17) and *C. krusei* BC34 (1.92 Abs/cm^2^ ± 0.41). It is relevant to note that all of these strains were isolated from children.

**Figure 2:**
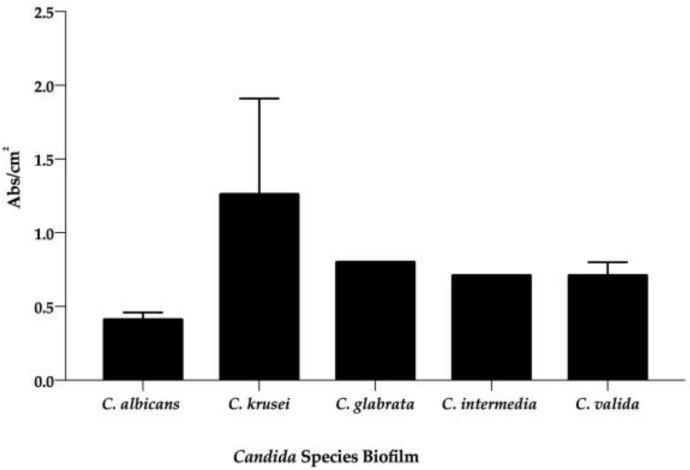
Average of in vitro biofilm (Abs/cm^2^) production from each species of *Candida*. The black bars are the mean of biofilm (Abs/cm^2^), which was quantified through staining with crystal violet (OD_570_) after 48 h of incubation. Each value is the average of three independent experiments conducted in triplicate.

Surprisingly, if we group the results based on the species of *Candida*, the highest biofilm former was also found to be *C. krusei* (Figure 2). On the other hand, the in vitro average of matrix production by the other *Candida* spp. (i.e., the average of all strain species) was similar (Figure 2). Hence, when comparing the biofilm formation among species of *Candida* from all groups (Figure 2), *C. krusei* ($\bar{x}$ = 0.94 Abs/cm^2^ ± 0.80) produced the highest quantity of biofilm biomass, followed by *C. glabrata* ($\bar{x}$ = 0.80 Abs/cm^2^), *C. intermedia* ($\bar{x}$ = 0.71 Abs/cm^2^)*,* and *C. valida* ($\bar{x}$ = 0.71 Abs/cm^2^ ± 0.13). Curiously, in this study, *C. albicans* ($\bar{x}$ = 0.42 Abs/cm^2^ ± 0.19) was the species presenting the lowest ability to form biofilms. On the other hand, these averages show no statistically significant differences.

**Figure 3:**
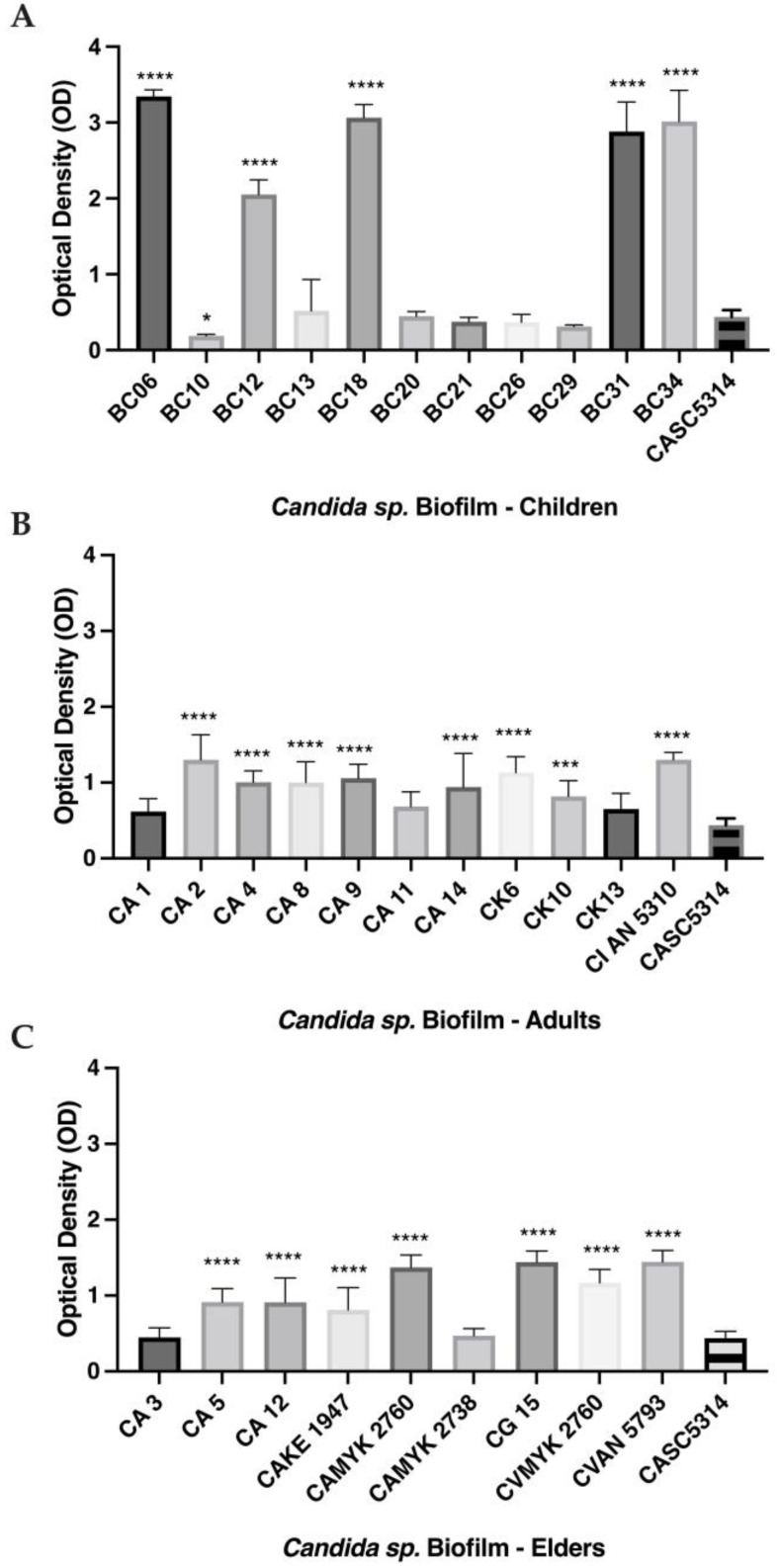
In vitro biofilm production of clinical isolates from children (**A**), adults (**B**), and elders (**C**). Biofilm was quantified through staining with crystal violet (OD_570_) after 48 h of incubation. The grey bars are the biofilm biomass of each strain. Each value is the average of three independent experiments conducted in triplicate. Error bars represent the mean and standard deviations. **** *p*  <  0.0001, *** *p*  <  0.001 and * *p*  <  0.05 as compared to biofilm formed by the reference strain (*C. albicans* SC5314). Note: CA—*C. albicans*; CK—*C. krusei*; CG—*C. glabrata*; CI—*C. intermedia*; CV—*C. valida*.

The biofilms of all clinical isolates were compared to the reference strain, *C. albicans* SC5314, since it presents a biofilm with high hyphal quantity and entanglement [8,10]. In the children’s group, we observed that BC06, BC12, BC18, BC31, BC34 (*p* > 0.01), and BC10 (*p* > 0.05) had a higher ability to produce biofilms (Figure 3A). For strains isolated from adults, all biofilms presented higher absorbance values than the reference (meaning bigger biofilm production) except for *C. albicans* CA1, *C. albicans* CA11, and *C. krusei* CK13 (Figure 3B). Similarly, in the elders’ group, most strains were stronger producers of biofilm when compared to *C. albicans* SC5314, excepting *C. albicans* CA3 and *C. albicans* CAMYK 2738 (Figure 3C). Among all *C. albicans* strains, *C. albicans* MYK 2760 was the strongest biofilm producer (0.76 Abs/cm^2^ ± 0.09) and the weakest were *C. albicans* BC29 (0.15 Abs/cm^2^ ± 0.02) and *C. albicans* CA3 (0.15 Abs/cm^2^ ± 0.03). On the other hand, the strain control, *C. albicans* SC5314, presented intermediary values (0.29 Abs/cm^2^ ± 0.21) (Figure 3).

### 2.3. Biofilm Matrix Composition

**Figure 4:**
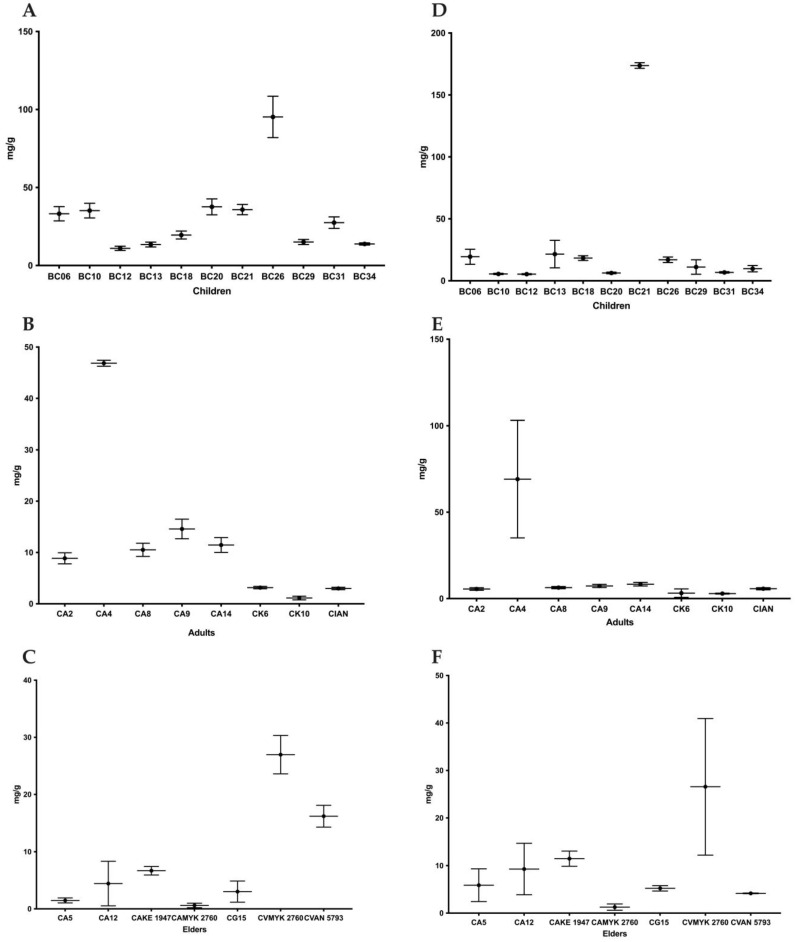
Average quantity of protein (**A**–**C**) and polysaccharides (**D**–**F**) isolated from a matrix of in vitro biofilm produced by clinical isolates from children, adults, and elders. Each value is the average (protein mg/g of biofilm) ± standard deviation (SD). Note: CA—*C. albicans*; CK—*C. krusei*; CG—*C. glabrata*; CI—*C. intermedia*; CV—*C. valida*.

**Figure 5:**
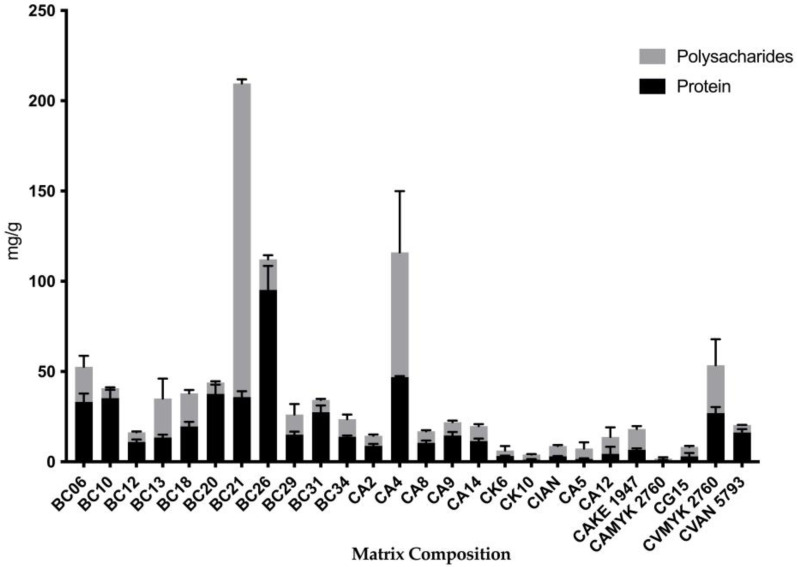
Proportion of protein (OD_562_) and polysaccharide (OD_490_) quantity from each strain from different ages. The gray bars are the polysaccharide level, and the black bars are the protein level of each strain. Each value is the average (mg/g of biofilm) of three independent experiments conducted in triplicate ± standard deviation (SD). Note: CA—*C. albicans*; CK—*C. krusei*; CG—*C. glabrata*; CI—*C. intermedia*; CV—*C. valida*.

For the study of biofilm composition, the polysaccharide content was determined using the phenol/sulfuric acid method and the protein content was determined with the BCA Kit (Figure 4 and Figure 5) [10]. The highest level of polysaccharides was observed in *C. glabrata* BC21 from the children’s group and the lowest in biofilm of *C. albicans* MYK 2760 biofilm from the adults’ group (Figure 4).

On the other hand, the quantities of proteins were the highest in the biofilms of *C. krusei* BC26 from the children’s group and the lowest in the matrix of *C. albicans* MYK 2760 biofilm from the adults’ group (Figure 4). In addition, the strains that had the highest polysaccharide levels (BC13 and BC21) also had more polysaccharide than protein in their extracellular matrices (Figure 5).

### 2.4. Effect of Antifungals against Biofilm Formation of Different Candida Species

In order to determine the susceptibility of biofilms to antifungals, fluconazole (1250 mg/L), voriconazole (800 mg/L), anidulafungin (2 mg/L), and amphotericin B (2 mg/L) were added at the start of the experiment. The goal was to evaluate the effect of these drugs on the development of the biofilms. These concentrations were carefully chosen, taking into account several previous studies conducted by our group with the same conditions (antifungal drugs used to treat matured biofilms of *Candida* spp.) [10,11,12,13,14,15].

**Figure 6:**
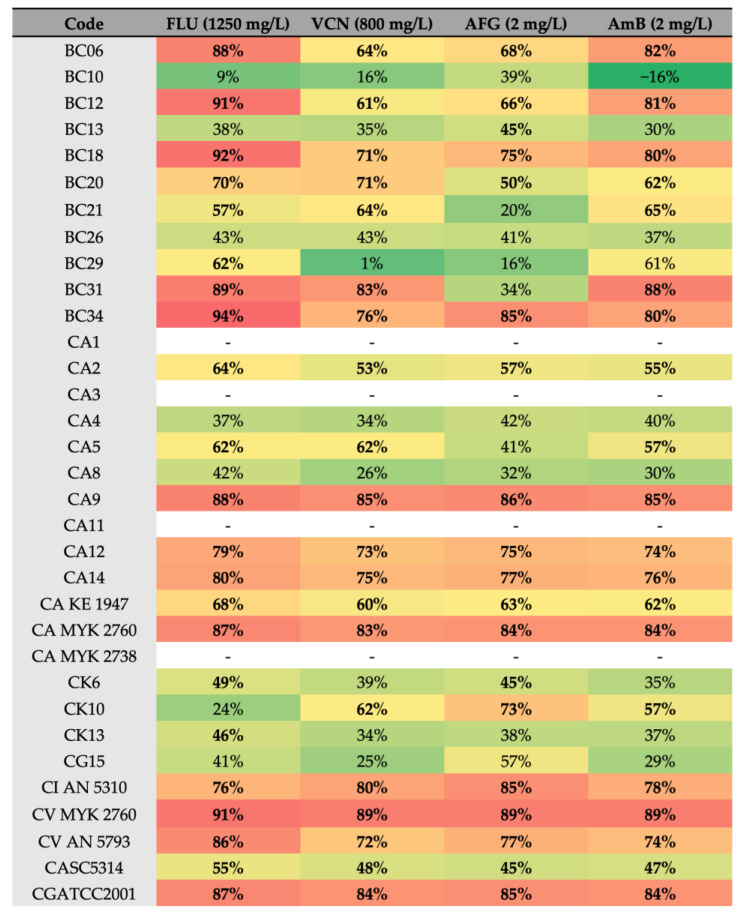
Heatmap of the reduction (%) of biofilm formation in the presence of antifungals. Red > 80%, orange > 65%, yellow > 50%, and green < 50% reduction. Note: FLU—Fluconazole, VCN—voriconazole, AFG—anidulafungin, AmB—amphotericin B. Bold: above 50%.

Figure 6 presents the percentage of biomass reduction in the presence of the different antifungals. Generally, all strains showed significant biomass reduction in the presence of antifungals. The exceptions were the biofilms of *C. glabrata* BC10, which presented a significant increase in total biomass values (*p* < 0.01) with amphotericin B (2 mg/L) when compared with the controls (biofilms without antifungals).

**Figure 7:**
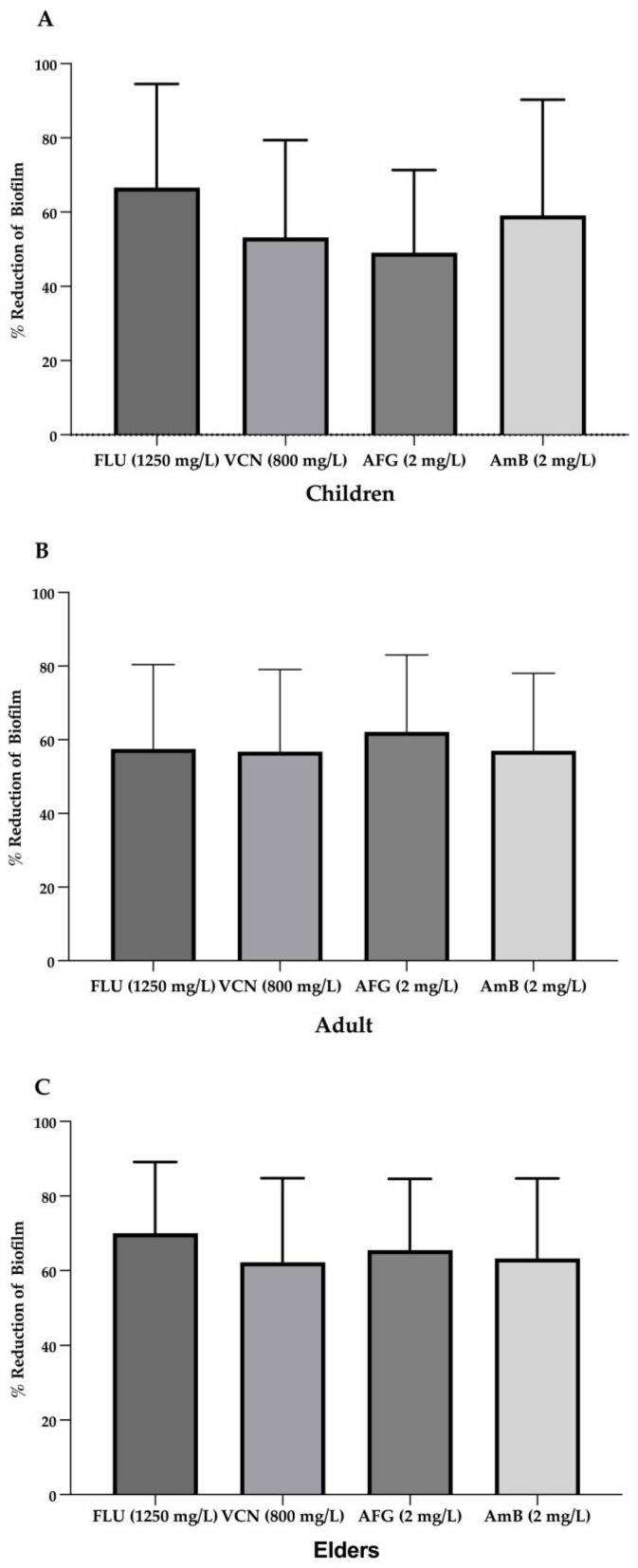
Average of the reduction (%) of biofilm formation in the presence of antifungals in children group (**A**), adult group (**B**) and elders’ group (**C**). Note: FLU—Fluconazole, VCN—voriconazole, AFG—anidulafungin, AmB—amphotericin B.

The lowest values of biofilm reduction were obtained for *C. albicans* BC29 treated with Vcn at 800 mg/L (Figure 6). Furthermore, strains isolated from children and elders showed greater susceptibility to Flu than strains isolated from adults (Figure 7).

### 2.5. Confocal Laser Scanning Microscopy

**Figure 8:**
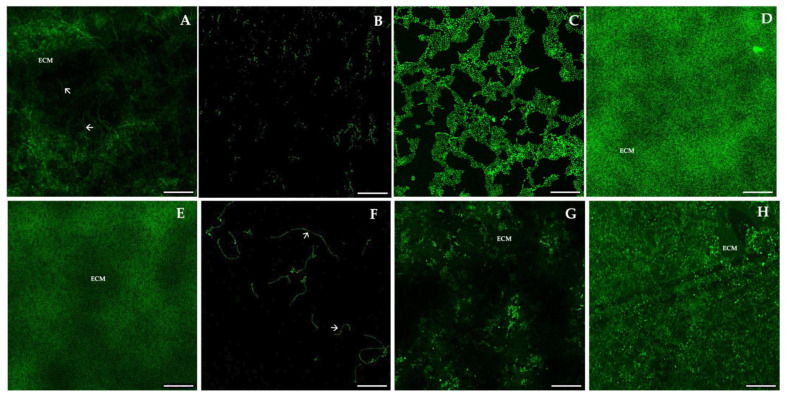
Representative images of confocal laser scanning microscopy (CLSM) of mature biofilms of *Candida* spp. from children, adults, and elders, analyzed by PNA-FISH using the *C. albicans* PNA probe. (**A**): Mature biofilm of *C. albicans* SC5314; (**B**): *C. glabrata* BC21; (**C**): *C. albicans* BC29; (**D**): *C. valida* AN5793; (**E**): *C. krusei* 6; (**F**): *C. albicans* CAMYK 2760; (**G**): *C. intermedia* AN5310; (**H**): *C. krusei* BC06. Magnification: 600×. Laser: 488 nm (ALEXA^®^-488). Arrows indicate hyphae form, and ECM means extracellular matrix. Scale bar: 100 μm.

Confocal scanning laser microscopy (CLSM) was performed in order to evaluate the organization and architecture of the biofilms. The images of the biofilms were presented individually or reconstructed in 3D projections (Figure 8, Panel A–I). In addition, vertical (x-z) sections or side views of the 3D reconstructed images were used to determine biofilm thickness and architecture.

*C. albicans* SC5314 (A) showed an expected heterogeneous architecture of a mature biofilm, with cells and hyphae embedded within the extracellular matrix (ECM). Differences between the biofilms formed by all species and strains are clearly visible in Figure 8, which is reflected in the architecture, the amount of extracellular polymeric substances (EPS), and the thickness. Panels H (*C. krusei* BC06) and G (*C. intermedia* AN5310) show biofilms covering the entire surface of the slide, similarly to A, but no hyphae cells are observed, as these species are non-dimorphic fungi. In panels B (*C. glabrata* BC21) and F (*C. albicans* CAMYK 2760), we can see microcolonies of predominant yeast forms visible (panel B) and yeast and hyphae forms (panel F), while panel D (*C. valida* AN5793) shows a mature biofilm with a lower quantity of EPS. In contrast, in panel E (*C. krusei* 6), there are cells in mature biofilms, enclosed in ECM, appearing as diffuse green fluorescence. Finally, *C. albicans* BC29 (C) biofilm shows yeast cells and hyphae as the control but not the formation of a uniformly flat surface.

## 3. Discussion

The genus *Candida* includes approximately 300 species, and it is a member of the healthy microbiota, asymptomatically colonizing several human tissues [16,17,18,19]. *Candida* represents one of the most common causes of systemic infection in hospitalized patients [20]. For hospitalized children, *Candida* spp. are the second most common pathogen identified in the setting of sepsis [21] and are the leading infectious cause of death in children with cancer or following an organ or hematopoietic stem cell transplant [21].

In oral microbiota, *Candida* spp. have high probability to infect other tissues, developing systemic infection [22,23]. *C. albicans* is still the most prevalent species in the oral cavity [18,21,24], but NCACs (e.g., *C. glabrata*, *C. tropicalis*, *C. parapsilosis*, and *C. krusei*) have increased [25]. The identification of *Candida* spp. present in oral mucosa is a critical issue for the successful treatment of infected patients. This is particularly important for species such as NCACs which have shown high antimicrobial resistance rates [21,22,24].

In this study, *C. krusei* was the predominant species in the oral cavities of children, which, curiously, in not in accordance with the current literature, which indicated that *C. albicans* is the predominant species [24]. However, it is relevant to note that there are limited studies including *Candida* spp. involving pediatric patients, because, typically, they involve healthy and/or adult patients [21,24]. Additionally, past studies that focused candidemia observed that NCACs were predominant in pediatric patients, and *C. tropicalis* was the most common species (50% of the infections) [26]. Previously, a multi-national study of pediatric candidiasis observed a higher proportion of *C. guilliermondii* and *C. krusei* in non-US sites [25]. In the same study, of the 449 *Candida* isolates recovered, 40% were *C. albicans*, and NCACs predominated in 60% of the cases [25]. Similarly, a prospective epidemiologic study of invasive candidiasis in children and neonates enrolled 196 pediatric and 25 neonatal patients with invasive candidiasis in different countries. It was observed that NCACs prevailed in the pediatric (56%) and neonatal (52%) age groups [27]. In this same study, a difference was also observed among antimicrobial responses. The antimicrobial treatment was successful in most of the children, but when *C. glabrata* was involved, there was a lower successful outcome rate (55%). The most-used antifungal therapies for pediatric and neonatal invasive candidiasis were fluconazole (21%), liposomal amphotericin B (20%), and micafungin (18%), and no significant differences were observed among the classes of antifungals [27]. Children are thought to be more susceptible to antimicrobials due to their reduced exposure and also to their shorter lifetimes and limited number of illnesses. However, we cannot put aside environmental exposure to drugs, which has been shown to have a critical role in selection pressure and the presence of multiresistant strains in volunteers’ flora [20,21].

Not surprisingly, as other studies have shown [1,2,16], our results indicated that, in adults and elders, *C. albicans* was the most common species, followed by *C. krusei*. Epidemiological studies have shown that, among NCACs, the predominant species in candidemia is *C. glabrata* in the USA, Northern Europe, and Australia, while *C. parapsilosis* is the most prevalent in Latin America, Southern Europe, and Asia [28].

The association between antimicrobial resistance and biofilm production ability is known [7,19,29,30,31,32,33,34]. Thus, biofilm characterization is essential in order to find therapeutic protocols capable of preventing and treating chronic infections. Patients with oral candidiasis are mostly treated with antifungal agents such as polyenes (e.g., AmB and nystatin) and azole derivatives (e.g., fluconazole, ketoconazole, clotrimazole, and miconazole) [35]. It has been reported that 20% of patients with oral candidiasis experience recurrence of infection and that around 30% of these recurrences are caused by different *Candida* strains than from the first episode of infection [3,35]. Usually, recurrent infections are correlated to biofilm formation [1,3,7,12,25,28,30,36].

In this study, the biofilms’ comparisons were made while taking into account *C. albicans* SC5314 biofilm, which has high hyphae quantity and entanglement [8,11]. The results showed that all of the strains were biofilm formers, regardless of the age group. Still, the characteristics of biofilms were different between age groups. The biofilm formed by isolates from children presented a higher biomass and a matrix composition richer in polysaccharides than the ones from adults. This it probably is due to the fact that NCACs were predominant in the children’s group. According to Kumari et al. [36], the total carbohydrate and protein content in the biofilm matrices was significantly higher in NCACs when compared with *C. albicans*. The matrix carbohydrate content was the highest in *C. tropicalis*, followed by *C. krusei*, and the lowest content was observed in *C. albicans*, which was predominant in the adult group. Rodrigues et al. also reported similar results in NCACs [8,36].

*C. albicans* are dimorphic fungi and hyphae are an important structural component of its biofilms [19,36]. The hyphae in biofilms contribute to the overall architectural stability of the biofilm and to adherence to normal biofilm development and maintenance [19]. Indeed, the genes associated to hyphal Efg1, Tec1, Ndt80, and Rob1 are also necessary for biofilm formation [19,20,29]. There is a direct relationship between *C. albicans* morphogenesis and exposure to antifungal agents [37,38]. It has been previously observed that fluconazole-susceptible yeasts show reduced hyphae production while resistant isolates were little affected by this antifungal agent [38,39,40]. Azoles are known to act by inhibiting the production of ergosterol, which is the main constituent of a fungal cell membrane [37,38,39]. These antifungal agents probably reduce the production of hyphae due to the increased surface area of hyphal cells compared to those of spherical forms. Previous work shows that alterations in the *ERG11* gene, which is responsible for ergosterol production, limit hyphae formation, presumably due to the lack of ergosterol [19,37,38,39]. In addition, treatment with azole leads to an accumulation of the sterol biosynthetic intermediate farnesyl pyrophosphate, which indirectly stimulates the overproduction of farnesol and is capable of inhibiting filamentous growth in *C. albicans* [7,39]. The development of efflux pumps by *Candida* spp. is the most frequent mechanism of azole resistance. Therefore, the two gene families which encode the efflux pump deserve to be highlighted. The *CDR1* and *CDR2* genes belong to the ATP-binding cassette superfamily, as do the *MDR1* genes of the key facilitator superfamily. Thus, increased expression levels of *CDR1*, *CDR2*, and *MDR1* in *C. albicans* cause resistance to fluconazole [41].

The presence polysaccharides in the extracellular matrix, such as β-1,3 glucan, β-1,6 glucan, and α-1,2-branched α-1,6 mannan, contribute to the antimicrobial resistance of *Candida* biofilms [34,42,43]. The mechanism of the drug–polysaccharide interaction was first described for *C. albicans* biofilm matrix and the azole drug, Flu [40]. The glucan and mannan components for the extracellular matrix form a complex that sequesters drugs, likely through non-covalent interactions [33]. Interestingly, there is a correlation between cell wall glucan and biofilm growth [40]. Moreover, the mechanisms of drug sequestration for other *Candida* spp., including *C. tropicalis, C. parapsilosis, C. glabrata*, and *C. auris*, were associated with presence of glucan [13,19,33].

In addition, the matrix polysaccharides of *Candida* biofilms also sequester antimicrobial drugs, for instance, AmB, anidulafungin, and flucytosine [13,42]. Furthermore, a higher level of biomass and polysaccharides can be associated with *FKS1, FKS2, BGL2*, and *XOG1* genes’ expression [13,43,44]. The importance of these genes is related to the delivery and production of the β-1,3 glucans matrix and to the matrix structure and adherence of biofilm cells to a surface, or in other words, the antimicrobial resistance phenotype [15,42].

All of the strains that presented a low ability to produce biofilm (O.D. inferior to 0,5) were not tested. We would like to emphasize the lack of a direct correlation between a high concentration of polysaccharide in the matrix and biofilm antifungal resistance. However, and surprisingly, most of the strains presenting high levels of polysaccharide in their biofilm matrix were less sensible to antifungal drugs (drug susceptibility lower than 70%) (e.g., strains BC21, CA4, and BC13; Figure 5 and Figure 6). Of this group, BC21 displayed the highest drug susceptibility. This probably occurred because it was isolated from a 1-year-old child, and exposure to antifungals—which promotes the selection of resistant microorganisms—is low at this age. A higher susceptibility of *C. albicans* strains to antifungal drugs, as compared to NCACs, was also observed in the adult group. No direct correlation could be made with other virulence factors not considered in this work.

Importantly, it was observed that the proportion of protein/polysaccharides was directly associated with the susceptibility of the *Candida* spp. to the antimicrobial drugs in the children’s group, except in strains with a higher level of polysaccharides, such as BC13 (*C. krusei*) and BC21 (*C. glabrata*). Still, in the adult group, a relationship between the matrix composition and antimicrobial sensibility was not observed, probably because adult microbiota present other virulence factors than matrix production, such as ergosterol biosynthesis [20,45]. This is, for example, the case for the biofilm resistance to Flu and AmB, which is associated with a significant decrease in total ergosterol content as well as changes in the levels of other sterols by the expression of *ERG* genes, which act as binding sites for antifungal molecules, inhibiting their binding to ergosterol [3,40,45,46].

Previously, our study on the adult strains (planktonic form) showed that half of the isolates presented resistance to 5′-fluorouracil and that almost 29% had resistance to Flu [16]. All isolates were sensible to AmB, and two samples had an intermediate profile to Vcn [16]. Hence, almost 82% of the collected samples showed resistance to at least one antifungal class, which is clinically remarkable [16]. In this study, in biofilm form, a reduction of biomass was observed in all strains treated with antifungal agents except BC10 (*C. glabrata*) cultivated with AmB. This difference might be associated to the tested concentrations of the antifungal drugs. In the present study, the concentrations of antifungals used were previously known to be effective against *Candida* biofilms.

Beforehand, our group showed that the minimum fungicidal concentration (MFC) for Flu and Vcn for *C. glabrata* ATCC 2001 was almost 200-fold and 4-fold higher than the MIC, respectively [12,13]. Also, Fonseca et al. [47] demonstrated that Flu at 1250 mg/L can be effective for biomass reduction (*p* < 0.01), even in the resistant strains, according to the MIC breakpoint of EUCAST [48]. When comparing Flu and Vcn, another study observed that *C. glabrata* biofilms were more susceptible to Vcn and, at 1000 mg/mL, the latter was effective in inhibiting the biofilms [12]. Curiously, there was an overexpression of the three *ERG* genes in the presence of both drugs and an increase of β-1,3 glucans in matrices [12]. Importantly, the use of Vcn is recommended for esophageal and oropharyngeal candidiasis in cases of fluconazole-refractory disease [12,49].

Similarly, Ramage et al. [50] described that AmB was up to 32 times less active on biofilm cells than on planktonic cells, with variable resistance being observed among the strains. At 2 mg/L, AmB reduced the biofilm biomass by 64.2% (*p* < 0.0001) and promoted a reduction of viable cells on *C. glabrata* ATCC2001 biofilms [15]. Other reports presented similar results for other *Candida* spp., demonstrating that AmB is still among the most effective drugs for the treatment of *Candida* spp. infections [15,37,51,52].

Anidulafungin was tested against the biofilm of reference strains and isolates of *C. albicans* and NCACs, and the inhibition of sessile cells was around 50% of the metabolic activity [53]. The effective concentration against biofilms of *C. albicans* ATCC10231, ATCC90028, *C. tropicalis* ATCC750, *C. albicans*, and *C. tropicalis* isolates was between 0.5 and 1 μg/ mL, more than five or six dilutions higher than their planktonic MICs [53]. In this study, it was used two times, and no resistance was observed. A previous study using caspofungin and micafungin against biofilm of *Candida* spp. showed that the concentration needed to eradicate the biofilm (MBEC) was five to six times higher than the MFC (planktonic cells). The MBEC was between 0.5–3 mg/L and 3.5–17 mg/L for caspofungin and micafungin, respectively [8].

In conclusion, children were more likely to be infected by NCACs when comparing with adults and elders. As a key factor, these NCACs produced biofilms richer in matrix components. This is a very relevant clinical finding, since stronger biofilms are directly linked with higher antifungal resistance (and, thus, therapeutic failure).

As a limitation of our study, we have no further information on the source of the samples (previous antibiotic therapy and co-morbidities), and the studied populations were heterogenous.

## 4. Materials and Methods

### 4.1. Collection and Identification of the Clinical Isolates

Some clinical isolates of *Candida* spp. (*n* = 31) were collected by a lab technician from the oral mucosa (swab from tongue or oral mucosa) of patients of the primary health care unit of Acarape city and were maintained at the laboratory of the Department of Microbiology of the University of International Integration of Afro-Brazilian Lusophony (UNILAB) in Brazil. Others were collected (following the same procedure) and maintained by the Department of Clinical Microbiology, from several wards of the Nitra Faculty Hospital in Slovakia. Samples were immediately stored and kept at −80 °C, until accurate identification by biomolecular methods. Then, all of the isolates were sent to the LEPABE laboratory at the University of Porto for the development of this work.

The study was conducted according to the guidelines of the Declaration of Helsinki and was approved by the Institutional Review Board (or Ethics Committee) of the Nitra Faculty Hospital in Slovakia (Code: 020322; Date: 2 March 2022). In Brazil, the study was preceded by the approval of the committee of ethics in research (CEP) under the number 4.432.501, following the ethical aspects of the resolution 466/12 and 510/16 of the Conselho Nacional de Saúde. All of the names and private information of patients were kept confidential.

The clinical isolates were coded as:

- Children: BC06, BC10, BC12, BC13, BC18, BC20, BC21, BC26, BC29, BC31, and BC34.
- Adults: CA1, CA2, CA4, CA8, CA9, CA11, CA14, CK6, CK10, and CIAN 5310.
- Elders: CA3, CA5, CA12, CAKE 1947, CAMYK 2760, CA MYK 2738, CG15, CVMYK 2760, and CVAN 5793.

Reference strains *Candida albicans* SC5314 and *Candida glabrata* ATCC2001 were acquired from the American Type Culture Collection.

In all cases, for routine identification, *Candida* isolates were grown in Sabouraud Dextrose Agar (SDA) (Merck, Darmstadt, Germany) under aerobic conditions for 24 h at 37 °C. The procedures for identification were performed by standard mycological methods at 30 °C for 48 h using chromogenic medium CHROMagar *Candida* (CHROMagar Microbiology, Paris, France) [14,15].

All samples from Slovakia have the patient data published in the article Černáková et al. [16]. Regarding the samples from the children, they were all clinically healthy.

### 4.2. Inoculum Preparation

*Candida* species were grown on SDA and incubated for 24 h at 37 °C. In order to prepare the inoculum, cells were then inoculated in SDB Sabouraud dextrose broth (SDB) (Merck, Darmstadt, Germany) and incubated for 18 h at 37 °C under agitation at 120 rpm. After incubation, the inoculum density was adjusted to 1 × 10^5^ cells/mL using a Neubauer chamber with RPMI-1640 (Sigma-Aldrich, St. Louis, MO, USA) [46].

### 4.3. Antifungal Drugs

Fluconazole, voriconazole, and anidulafungin were provided by Pfizer^®^ (New York, NY, USA)—Pfizer) in their pure form. Amphotericin came from Sigma^®^ (Sigma-Aldrich, Buffalo, NY, USA). Aliquots of 5000 mg/L of fluconazole (Flu) and voriconazole (Vcn) and 40 mg/L of amphotericin B (AmB) and anidulafungin (Afg) were prepared using dimethyl sulfoxide (DMSO) for all drugs. The final concentrations used were prepared in RPMI-1640 (Sigma-Aldrich, St. Louis, MO, USA). For DMSO, 1% was also used as a control.

### 4.4. Biofilm Formation and Crystal Violet Assay

The characterization of biofilm formation by *Candida* spp. was performed according to Rodrigues et al. [13]. Briefly, after the inoculum, a total of 100 μL of each strain inoculum was transferred to each well of the 96-well micro-plate, and then 100 μL of RPMI-1640 was added, supplemented or not with antifungals for 48 h at 37 °C. The antifungals tested were fluconazole (1250 mg/L), voriconazole (800 mg/L), anidulafungin (caspofungin/micafungin) (2 mg/L), and amphotericin B (2 mg/L). It is relevant to indicate that these concentrations were chosen, according to previous studies of our group, as the minimum concentrations to eradicate the biofilm (MBEC) in several *Candida* spp. [8,10,11,12,13,14,15]. Wells containing only culture medium without inoculum were used as negative control.

After incubation, the biofilm biomasses were analyzed by the crystal violet (CV) assay. For this, the supernatant was carefully aspirated, and the wells were washed twice with 200 μL PBS (phosphate-buffered saline, 0.1 M, pH = 7.2). Subsequently, biofilms were fixed by 100% (*v/v*) methanol, 200 μL/well, for 20 min. After drying, the supernatants were aspirated, and 200 μL of 1% (*v/v* aqueous CV was added to each well (Sigma–Aldrich, St. Louis, MO, USA). After 5 min, the dye solution was aspirated, and the wells were washed twice with sterile distilled water. Subsequently, 200 μL of a 33% acetic acid solution was added to each well and immediately transferred to a new 96-well plate. Finally, the plates were read at 570 nm (FLUOStar Omega Plate Reader, BMG LABTECH, Ortenberg, Germany) [8]. The cut-off optical density (ODc) was defined as three standard deviations above the mean OD of the negative control, and the strains were classified as follows: OD ≤ Odc = no biofilm producer; Odc < OD ≤ 2 × Odc = weak biofilm producer; 2 × Odc < OD ≤ 4 × Odc = moderate biofilm producer; and 4 × Odc < OD = strong biofilm producer [10].

### 4.5. Quantification of Matrix Polysaccharides

The quantification of polysaccharides was performed by the phenol-sulfuric acid method, according to Rodrigues et al. [8]. Briefly, the biofilm matrix was collected after the 48 h incubation period, then the biofilms were scraped (two times, using a 100 uL tip) from the 24-well plates, resuspended in ultra-pure water, sonicated for 60 s (Sonopuls HD 2200, Bandelin, Berlin, Germany), vortexed for 2 min, and centrifuged at 2898× *g* (4000 rpm) for 8 min. The supernatant was sterilized with a 0.22 μm filter membrane [10]. In a small test tube with 0.5 mL of supernatant (matrix), 0.5 mL of phenol (50 g/L) and 2.5 mL of sulfuric acid (95–97%) were added. The mixture was vortexed and allowed to stand for 15 min at room temperature. Then, the absorbance was measured at 490 nm by a multilabel plate reader, and the results were expressed as absorbance [10]. A blank without glucose was set for each assay. The quantity of polysaccharides was extrapolated from a standard curve made with standard glucose concentrations. The quantity of polysaccharides was then normalized by dry weight of biofilm (mg polysaccharides/g biofilm) [10].

### 4.6. Quantification of Matrix Protein

The extracted biofilm matrix (25 μL) was transferred to 96-well plates (triplicate) and 200 μL of reagent mixture of Novagen^®^ BCA Protein Assay Kit (Merck KGaA, Darmstadt, Germany) were added to each well. The solution was homogenized by vortex and incubated for 30 min at 37 °C. Then, the absorbance at 562 nm was determined using PBS as control. The amount of protein was extrapolated from a standard curve performed with standard BSA concentrations. The amount of protein was then normalized by dry weight of biofilm (mg protein/g biofilm) [8].

### 4.7. Assessment of the Spatial Arrangement of Candida *spp.* Cells in Biofilms by CLSM

A specific 23S rRNA PNA probe developed and optimized by our group was used for *Candida* spp. detection: 5′-Alexa488-OO-CACCCACAAAATCAA-3′ (melting temperature: 75.69 °C; specificity: 96.04%; sensibility: 84.79%). The probe was synthesized (Panagene, Daejoen, Republic of Korea), attached to the Alexa^®^-488 fluorochrome, and tested with *C. albicans* SC5314.

For the biofilm spatial organization, the PNA-FISH were performed directly in the coverslip Thermanox™, plastic slices coated by poly-L-lysine (to enhance cells adhesion) on which biofilms were grown, over the course of 48 h. Briefly, *Candida* spp. biofilms were formed on commercially available, presterilized, polystyrene, 12-well microtiter plates. In each well, there was a Thermanox™ coverslip (5 mm) for the development and growth of the biofilms. After the period of biofilm formation, all of the medium was aspired, and the biofilms were washed once with PBS to remove non-adherent cells (and to avoid biofilm loss). Then, biofilms were fixed in 4% (*w*/*v*) paraformaldehyde (Sigma-Aldrich, St Louis, MO, USA) followed by 50% (*v*/*v*) ethanol for 30 min at −20 °C and were incubated with PNA Probe at 54 °C. After a 30 min incubation period in the dark, the sample was observed by CLSM (LSM 710, Carl Zeiss, Germany). Image acquisition was performed using a 60× oil-immersion objective (60×/1.2 W) and the 488 nm laser line. Z-stacks with 1 µm Z-steps were collected. All microscope settings were identical among the analyzed groups. Zeiss Zen software was used for confocal image acquisition and processing, and ImageJ software was used for analysis.

### 4.8. Statistical Analysis

The experimental data were evaluated by GraphPad Prism v.9.1.1 software (San Diego, CA, USA). The data were analyzed using one-way ANOVA followed by Dunnett’s test. In all cases, statistical significance was set as *p* < 0.05. Data are presented as the mean ± standard deviation (SD). All experiments were performed three times independently, in triplicate.

## 5. Conclusions

The resistance of *Candida* spp. biofilms to different antifungal agents is multifactorial [10,30,36]. Features of virulence, such as membrane and cell wall barriers, dimorphism, the signal transduction pathway, proteins related to stress tolerance, hydrolytic enzymes (e.g., proteases, lipases, and hemolysins), and toxin production should be studied in the future in order to better understand the isolated strains as well as the pathogenesis of the genus *Candida* [6].

Here we show that the oral biofilm displays different species of *Candida* in terms of quantity, matrix composition, and, consequently, susceptibility to antifungal drugs, depending, for instance, on the age group. These might be related to the maturity of the immune system and metabolism, but also to habits such as diet, physical activity, smoking, antibiotic use, and associated diseases. The results of this study might help us to better control these infections and to choose adequate therapy depending on the species, immunity state, and age. The present work opens the door to new studies that can deepen our understanding of virulence factors and pathogenicity in *Candida* spp. among different ages, allowing for the reduction of resistant strains and promoting more efficient treatments.

## Appendix A

**Table A1:** Demographic data associated with biofilm production for each strain.

Strain Code	Species	O.D._570_	Age	Gender
BC06	*C. krusei*	3.34 ± 0.08	7	Female
BC10	*C. glabrata*	0.19 ± 0.02	4	Female
BC12	*C. krusei*	2.05 ± 0.19	3	Male
BC13	*C. krusei*	0.52 ± 0.41	6	Female
BC18	*C. krusei*	3.06 ± 0.17	2	Female
BC20	*C. krusei*	0.45 ± 0.06	3	Male
BC21	*C. glabrata*	0.37 ± 0.08	1	Female
BC26	*C. krusei*	0.37 ± 0.10	1	Female
BC29	*C. albicans*	0.31 ± 0.02	5	Female
BC31	*C. krusei*	2.88 ± 0.39	5	Female
BC34	*C. krusei*	3.01 ± 0.41	6	Female
CA1	*C. albicans*	0.62 ± 0.17	14	Male
CA2	*C. albicans*	1.25 ± 0.36	46	Female
CA3	*C. albicans*	0.45 ± 0.12	66	Male
CA4	*C. albicans*	1.01± 0.14	55	Male
CA5	*C. albicans*	0.92 ± 0.17	70	Male
CA8	*C. albicans*	1.00 ± 0.27	34	Male
CA9	*C. albicans*	1.06 ± 0.18	20	Female
CA11	*C. albicans*	0.68 ± 0.19	29	Female
CA12	*C. albicans*	0.91 ± 0.32	72	Female
CA14	*C. albicans*	0.94 ± 0.44	28	Male
CA KE 1947	*C. albicans*	0.81 ± 0.29	66	Female
CA MYK 2760	*C. albicans*	1.37 ± 0.16	74	Male
CA MYK 2738	*C. albicans*	0.47± 0.09	71	Male
CK6	*C. krusei*	1.13 ± 0.20	26	Male
CK10	*C. krusei*	0.82 ± 0.20	56	Male
CK13	*C. krusei*	0.65 ± 0.20	49	Male
CI AN 5310	*C. intermedia*	1.30 ± 0.09	37	Female
CG15	*C. glabrata*	1.44 ± 0.14	68	Male
CV MYK 2760	*C. valida*	1.17 ± 0.17	78	Female
CV AN 5793	*C. valida*	1.45 ± 0.14	70	Male

Note: CA—*C. albicans*; CK—*C. krusei*; CG—*C. glabrata*; CI—*C. intermedia*; CV—*C. valida*.

## Appendix B

**Table A2:** Matrix composition of *Candida* spp. biofilms isolated from children, including mean values of protein quantity (mg/g of biofilm) and mean values of polysaccharide quantity (mg/g of biofilm) ± standard deviation (SD).

	BC06	BC10	BC12	BC13	BC18	BC20	BC21	BC26	BC29	BC31	BC34	CASC 5314
Protein (mg/g)	33.19 ± 4.60	35.22 ± 4.70	11.01 ± 1.35	13.45 ± 1.59	19.55 ± 2.55	37.63 ± 5.10	35.84 ± 3.30	95.26 ± 13.27	15.05 ± 1.63	27.48 ± 3.70	13.82 ± 0.70	1.24 ± 0.05
Polysaccharides (mg/g)	19.43 ± 6.07	5.55 ± 0.49	5.36 ± 0.44	21.57 ± 11.11	18.34 ± 1.87	6.34 ± 0.64	173.78 ± 2.26	16.96 ± 2.24	11.13 ± 5.86	6.80 ± 0.61	9.79 ± 2.54	0.03 ± 0.05

Note: CA—*C. albicans*.

**Table A3:** Matrix composition of *Candida* spp. biofilms isolated from the adult group, including mean values of protein quantity (mg/g of biofilm) and mean values of polysaccharide quantity (mg/g of biofilm) ± standard deviation (SD).

	CA2	CA4	CA8	CA9	CA14	CK6	CK10	CIAN 5310	CASC 5314
Protein (mg/g)	8.87 ± 1.08	46.86 ± 0.58	10.52 ± 1.29	14.59 ± 1.91	11.46 ± 1.44	3.16 ± 0.23	1.13 ± 0.36	2.99 ± 0.24	1.24 ± 0.05
Polysaccharides (mg/g)	5.54 ± 0.74	69.13 ± 33.98	6.36 ± 0.66	7.31 ± 0.90	8.34 ± 1.04	3.15 ± 2.45	2.91 ± 0.26	5.70 ± 0.62	0.03 ± 0.05

Note: CA—*C. albicans*, CK—*C. krusei*; CI—*C. intermedia*.

**Table A4:** Matrix composition of *Candida* spp. biofilms isolated from elders, including mean values of protein quantity (mg/g of biofilm) and mean values of polysaccharide quantity (mg/g of biofilm) ± standard deviation (SD).

	CA5	CA12	CAKE 1947	CAMYK 2760	CG15	CVMYK 2760	CVAN 5793	CASC 5314
Protein (mg/g)	1.48 ± 0.44	4.43 ± 3.90	6.68 ± 0.75	0.60 ± 0.39	3.02 ± 1.85	26.97 ± 3.36	16.19 ± 1.91	1.24 ± 0.05
Polysaccharides (mg/g)	5.88 ± 3.45	9.28 ± 5.42	11.47 ± 1.59	1.27 ± 0.66	5.22 ± 0.56	26.58 ± 14.36	4.16 ± 0.07	0.03 ± 0.05

Note: CA—*C. albicans*, CK—*C. krusei*; CI—*C. intermedia*; CG—*C. glabrata*; CV—*C. valida*.
